# Bariatric surgery complicating the treatment of choice for Sporotrichosis: report of two cases^☆^

**DOI:** 10.1016/j.abd.2024.07.005

**Published:** 2024-11-09

**Authors:** Maria Eduarda Resende Melo, Melissa Orzechowski Xavier, Rossana Patricia Basso, Karine Ortiz Sanchotene, Fabiana Fedatto Bernardon, Vanice Rodrigues Poester

**Affiliations:** a Mycology Laboratory, Faculty of Medicine, Universidade Federal do Rio Grande, Rio Grande, RS, Brazil; b Postgraduate Program in Health Sciences, Faculty of Medicine, Universidade Federal do Rio Grande, Rio Grande, RS, Brazil; c Hospital Universitário Dr. Miguel Riet Corrêa Jr., Universidade Federal do Rio Grande, Empresa Brasileira de Serviços Hospitalares, Rio Grande, RS, Brazil

*Dear Editor*,

Sporotrichosis caused by *Sporothrix brasiliensis* (*S. brasiliensis*) is currently a severe public health problem in Brazil, with thousands of cases described in almost all Brazilian states.^1^ The first choice of drug for sporotrichosis treatment is Itraconazole (ITZ), showing a rate of ∼90% cure in human patients. Terbinafine (TER) is the second-line treatment, indicated for therapeutic failures or when ITZ is counter-indicated.^2^ Data regarding the use of antifungal drugs in patients undergoing Roux-en-Y Gastric Bypass surgery (RYGB) is scarce in scientific literature,^3^ thus, we aim to report two cases of cat-transmitted sporotrichosis in post-bariatric surgery patients in southern Brazil.

The first patient was a 62-year-old woman who first presented to the Brazilian Public Health Service (SBPHC) in 2022 with ulcerated lesions on her left upper limb. Her probable diagnosis of lymphocutaneous sporotrichosis was established clinically and epidemiologically by the characteristics of her lesions, combined with her report of contact with a domestic cat with proven sporotrichosis. Treatment with ITZ 100 mg (12/12 hours) was initiated. During the following two weeks, the patient reported a worsening of the skin lesions and the appearance of new lesions on other anatomical (erythematous macules and nodules on lower limbs and right upper limb, and pustular lesions on the cervical region). Erythematous nodular lesions and arthralgia were compatible with the clinical manifestation of erythema nodosum, a hypersensitivity reaction of sporotrichosis. At this moment, she searched for private dermatological care and was referred to the Specialized Reference Service (SRS) of the University Hospital (UH) from the *Universidade Federal do Rio Grande/Empresa Brasileira de Serviços Hospitalares* (FURG/Ebserh).^4^ At the SRS, on September 2022, the patient reported a history of RYGB surgery performed in 2021. Due to her history of non-response to the first-choice treatment, and given that the analysis of ITZ plasmatic levels was not available in our service, a therapeutic approach was changed to TER 250 mg (12/12 hours). The patient remained on the therapy for 140 days, showing total regression of the lesions after this period (Table 1 and Fig. 1).

**Table 1:** Clinical-epidemiological data regarding two cat-transmitted sporotrichosis cases in patients with a history of Roux-en-Y bariatric surgery reported in Southern Brazil.

	Case #1	Case #2
**Type of Diagnosis/Date**	Probable 2022	Probable 2023
**Clinical Apresentation**	Lymphocutaneous	Lymphocutaneous
**Site and Type of Lesions**	Ulcerous-Crusted on upper and lower limbs and nodular on anterior cervical region + HR^a^	Nodular on rigth hand
**Date of Roux-en-Y Bariatric Surgery**	3 years before	5 years before
**Initial Treatment**	ITZ 100 mg (12/12 h)	ITZ 100 mg (12/12 h)
**Modified Treatment**	TER 250 mg (12/12 h)	TER 250 mg/day
**Time of Treatment (only modified)**	140 days	106 days

a Hypersensitivity Reaction (erythema nodosum together with arthralgia).

**Figure 1:**
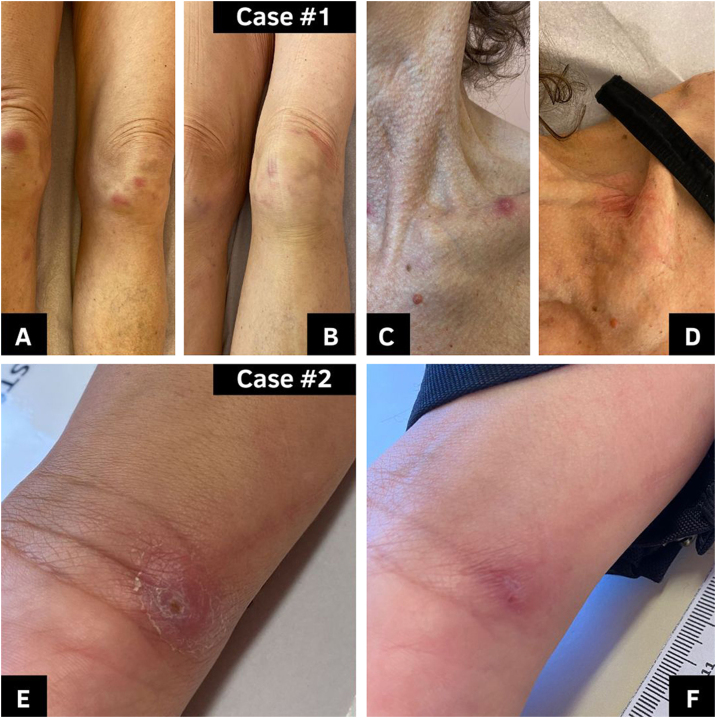
Two cases of cat-transmitted sporotrichosis in patients with a history of Roux-en-Y bariatric surgery reported in Southern Brazil. #Case 1: (A and C) Lesions on lower limbs and in cervical region, respectively, in the diagnosis (16/09/2022). (B and D) Lesions after 30 days of treatment (22/09/2022). #Case 2 (E) Lesion in the diagnosis (27/06/2023). (F) Lesion after 106 days of treatment (28/09/2023).

The second patient was a 48-year-old female with a history of RYGB surgery in 2019. She was admitted to the SRS in June 2023 with a nodular lesion on her right hand, compatible with a lymphocutaneous presentation of sporotrichosis (Fig. 1). She reported a previous scratch from a domestic cat with proven sporotrichosis, followed by two previous visits to SBPHC, where she has been prescribed antibacterial (azithromycin or cephalexin) and nonsteroidal anti-inflammatory drugs. She also reported that she had been using antifungal therapy (ITZ 100 mg; 12/12 hours) for 14 days on her own without improvement of the lesions. ITZ was discontinued and TER 250 mg/day was prescribed. Cure of sporotrichosis was achieved with 106 days of treatment.

We report two cases of therapeutic failure using ITZ in bariatric patients in Southern Brazil.^2^ These are the first reports of this emerging fungal infection in RYGB patients in a hyperendemic area where zoonotic sporotrichosis has been described since the 1990s.^1^ Our report also contributes to documenting another case of hypersensitivity reaction in the Southern region of Brazil. Indeed, hypersensitivity reactions are a growing manifestation in Brazilian territory, associated with the zoonotic epidemiology of sporotrichosis.^5^, ^6^

We assume that ITZ therapy failure occurred due to the RYGB technique on the Gastrointestinal Tract (GIT), which resulted in disabsorptive restriction by modifying the anatomical and physiological structure of the GITs. In fact, RYGB had a direct impact on the pharmacodynamics and pharmacokinetics of orally ingested drugs. The resulting decrease in gastric motility, volume, and bile secretion leads to a more alkaline pH and reduced first-pass metabolism.^7^ For ITZ, the lack of activity is due to its lipophilic nature, which requires ionization and absorption at a low pH (1‒3).^8^ Therefore, in patients with RYGB, the use of ITZ should be monitored through therapeutic drug monitoring by measuring the plasma concentration of the drug.^9^

Our reports add data on RYGB bariatric surgery patients, who developed sporotrichosis, showing the importance of been aware of the possibility of therapeutic failures with the drug of choice for this mycosis.

## Financial support

None declared.

## Authors’ contributions

Maria Eduarda Resende Melo: Drafting and editing of the manuscript; collection, analysis and interpretation of data; critical review of the manuscript.

Melissa Orzechowski Xavier: Drafting and editing of the manuscript.

Rossana Patricia Basso: Collection, analysis and interpretation of data; methodology.

Karine Ortiz Sanchotene: Collection, analysis and interpretation of data.

Fabiana Fedatto Bernardon: Methodology.

Vanice Rodrigues Poester: Drafting and editing of the manuscript; critical review of the manuscript.

## Conflicts of interest

None declared.
